# The choice of time scales in survival analysis has implications: calendar time versus patients’ time-to-event

**DOI:** 10.1186/s12874-026-02884-3

**Published:** 2026-05-20

**Authors:** Judith Vilsmeier, Gisela Büchele, Martin Rehm, Theresa Unseld, Dietrich Rothenbacher, Jan Beyersmann

**Affiliations:** 1 Institute of Statistics, Ulm University, Ulm, Germany; 2 Institute of Epidemiology and Medical Biometry, Ulm University, Ulm, Germany

**Keywords:** Cox proportional hazards model, Matching, Poisson regression, Real-time approach, Time scales

## Abstract

**Background:**

In the EvaCoM project, a retrospective cohort study of Germany-wide health insurance data of 29607 geriatric patients with fractures, the use of different time scales led to contradicting estimates in the Cox proportional hazards model. Here, the impact of quality audit, resulting in certification, of healthcare providers was of interest, with possible time scales being calendar time in which the audit acts or a patient’s time-to-event. For the analysis the patients were divided into groups depending on whether the certification occurred before or after the patient’s hospitalization, which adds a temporal component to the intervention (treatment in a certified hospital or not). A Cox model in calendar time found a harmful association of the audit with mortality (hazard ratio (HR) 1.13; 95% CI [1.04-1.23]), but in “time since hospital admission” no significant association was found (HR 1.04 [0.99-1.10]).

**Methods:**

To investigate these contradictions, the hazards in “time since hospital admission” are parametrically approximated with piecewise linear functions and transformed to calendar time by deriving patient-specific hazards based on their hospital admission date. A Poisson regression with both time scales simultaneously is used to investigate the contradictory results further. Matching is used to analyse the association in calendar time without using an additional time scale.

**Results:**

The analysis of the approximated and transformed hazards leads to the conclusion that using calendar time causes a structural bias, because, in calendar time, patients of the pre-certification group enter the study earlier than those in the post-certification group. In combination with time-dependent and decreasing hazards, this leads to an artificial increase of the hazard ratio. A Poisson regression using both time scales (HR 1.00 [0.92-1.08]) and a Cox regression on matched data (HR 0.99 [0.90-1.09]) confirmed this result, finding no significant association between audits and mortality.

**Conclusions:**

In EvaCoM, the estimation of significant association in calendar time is a consequence of a structural bias caused by patients being shifted in calendar time and not a true harmful effect. The choice of time scale may severely impact the results. We present methods to disentangle the effects of different time scales.

**Supplementary Information:**

The online version contains supplementary material available at 10.1186/s12874-026-02884-3.

## Background

Our motivation comes from a time scale issue, which occurred in the EvaCoM project. In this cohort study, the association of treatment in certified geriatric trauma centres with patients’ outcomes is being evaluated in a retrospective Germany-wide health claims data analysis [1]. Two time scales were of interest: the patient’s time-to-event, with hospital admission as the origin, and calendar time, i.e., “real” time [2]. The latter was of interest because it controls for time-varying factors affecting a hospital, such as seasonal factors. However, a Cox model with “time since hospital admission” as the underlying time scale found no significant association of treatment in certified trauma centres with mortality, but a Cox model in calendar time found a significant harmful association. These contradictory results stem from a structural bias caused by the use of calendar time, as in this time scale patients of the pre-certification group enter the study earlier than those in the post-certification group. In combination with time-dependent and decreasing hazards, this leads to an artificial increase of the hazard ratio.

That the use of different time scales can lead to contradicting results has previously been documented [3]. In time-to-event analysis the choice of time scale affects how probabilities vary over time and how to interpret estimates [3]. This choice is equivalent to choosing a time origin, a natural choice being the moment at which individuals start to be at risk for the event of interest, which may coincide with study entry [4]. However, if more than one time scale is assumed to influence probabilities, it might not be clear which time scale is most appropriate, as in the EvaCoM project.

This paper aims to explain how and why contradictions can arise when using different time scales, using the example of the EvaCoM project, and subsequently help to understand the impact that the choice of time scale can have. Since the focus of this paper is the methodological analysis of a structural problem, the reported analyses are univariate. Further adjusting the models to evaluate the association with the trauma centre is called for in practice, as the characteristics of patient populations of trauma centres differ from those of other hospitals [5]. Because the phenomenon of contradictory results still occurred when the models were adjusted for potential confounders, the observed differences in significance are not considered to stem from confounding issues, and we have chosen to focus on the univariate analyses for ease of presentation. The Methods section provides an overview of the EvaCoM project and its data, the certification of geriatric trauma centres, time scales and time-to-event methods used in this paper. The Impact of the time scale in the EvaCoM project section explains the cause of the contradicting results in the EvaCoM project. The Resolving the estimation of inflated hazard ratio in calendar time section introduces both matching and regression models that incorporate both time scales as a solution for resolving these contradicting results.

## Methods

 The Health claims data from Germany section describes the motivating study and the certification process. The time scales “time since hospital admission” and calendar time section introduces the two time scales used in this paper and the Time-to-event analysis section provides a short overview of time-to-event analysis. The Parametric approximation of hazards and switching between time scales section describes a parametric approximation of hazards in one time scale and their subsequent transformation to the other time scale. The Cox proportional hazards model section introduces the Cox proportional hazards model, as well as regression models that incorporate both time scales and matching.

### Health claims data from Germany

The EvaCoM project aims to investigate differences between treatment concepts for geriatric patients with specific bone fractures [1]. Since bone fractures seriously impact the health and quality of life of elderly individuals, special care and expertise is needed when treating geriatric patients. Such specialized treatment is provided in hospitals certified as geriatric trauma centres [6].

The certification concept was developed by the German Society of Traumatology (Deutsche Gesellschaft für Unfallchirurgie, DGU) and the German Society of Geriatrics (Deutsche Gesellschaft für Geriatrie, DGG). The DGU commissions an independent external certification body to conduct the certification process. Following a final positive evaluation of a checklist and documentation submitted for audit preparation, hospitals receive formal feedback from the certification body. In order to achieve certification, hospitals must comply with defined standard operating procedures for managing osteoporosis.

A core requirement of certification is the implementation of orthogeriatric co-management. This involves an interdisciplinary team of geriatricians and surgeons making at least two weekly visits to enable early geriatric rehabilitation to become a central component of patient care. Additionally, structured interdisciplinary care pathways must be established to manage delirium, diagnose and treat osteoporosis and malnutrition, and systematically identify patients requiring a preoperative geriatric assessment.

The EvaCoM project is a retrospective cohort study of nationwide health claims data of 29607 geriatric patients from 154 hospitals, which were certified from 2013 to 2021. The data were provided by the Research Institute of the AOK (Wissenschaftliches Institut der AOK, WIdO, Berlin, Germany). To investigate differences between treatment concepts, the association between certification to a trauma centre and time to death in different time scales was analysed.

Each patient was observed for a maximum of 180 days after hospital admission. To compare orthogeriatric co-management with standard treatment applied before certification, patients were divided into three groups depending on whether the hospital was certified before, during or after the patient’s hospitalization. The “Pre” group included patients admitted to the hospital up to 180 days before the certification date. The “Transitional” group included patients admitted between 180 days before certification and 180 days after certification, and the “Post” group included patients admitted to a hospital 180 days after certification or later. The group definitions thus ensured that the pre-, transitional-, and post-certification phases were strictly separated. The motivation for the “transitional” phase was that, as a prerequisite to certification, we expect an adaptation of the hospital procedures to start before the actual certification date. Additionally, it may take further time after the certification date until the procedures are fully established.

### The time scales “time since hospital admission” and calendar time

In this paper, we will discuss two time scales, the first being “time since hospital admission” and the second being calendar time. The origin (time “zero”) of the time scale “time since hospital admission” is the patient’s hospital admission, and time is counted in days from this origin until the patient has an event or is censored.

For calendar time, January 1^st^, 1970, was chosen as the origin, and time was also counted in days. Therefore, if, for example, a patient was admitted to a hospital on January 1^st^, 2015, and had an event on January 15^th^, 2015, he was admitted at $t=0$ on the time scale “time since hospital admission” and had the event at $t=14$. In calendar time, on the other hand, the patient was admitted to the hospital at $c=16436$, and the event occurred at $c=16450$. Here and in the following, we will use *t* to denote time since hospitalization and *c* to denote calendar time.

Consequently, in calendar time, patients must be analysed as being left-truncated (with entries after time zero), as they enter the study at different time points in this time scale [7]. In contrast, for “time since hospital admission”, a left-truncated analysis is not needed, as in this time scale all patients are under observation at time zero (hospital admission).

### Time-to-event analysis

In time-to-event analysis, the time $T \in (0,\infty )$ until an event occurs is of interest. *T* is subject to right-censoring if an event is not observed, i.e., if $T> C$, with *C* denoting the time until censoring.

The hazard in “time since hospital admission” is

1 $$\begin{aligned} \alpha (t) := \lim _{\Delta t \searrow 0} \frac{P(T \in [t,t+\Delta t) \ | \ T \ge t)}{\Delta t}, \end{aligned}$$

assuming the limit exists. In words, $\alpha (t) \textrm{d}t$ is the probability of having an event in the small time interval $[t,t + \textrm{d}t)$, given that no event occurred before. The hazard in calendar time can be defined analogously. Note, however, that in calendar time, there is no common time zero at which duration until the event starts. The cumulative hazard is

2 $$\begin{aligned} A(t):=\int _{0}^{t}\alpha (u)du. \end{aligned}$$

An estimator for the cumulative hazard is the Nelson-Aalen estimator

3 $$\begin{aligned} \hat{A}(t) = \sum \limits _{u \le t}\frac{\Delta N(u)}{Y(u)}, \end{aligned}$$

where *Y*(*u*) is the number of individuals at risk just before *u* and $\Delta N(u)$ is the number of events observed at *u*. The sum is over all unique observed event times $u \le t$.

On the time scale “time since hospital admission”, there is a common time zero, and the survival function $S(t) := P(T>t)$ is the probability that an individual has not yet had an event at time *t*. It holds that $S(t)= \exp (-A(t))$. The survival probability can be estimated with the Kaplan-Meier estimator

$$\begin{aligned} \hat{\textrm{S}}(t) = \prod _{u \le t}\left( 1-\frac{\Delta N(u)}{Y(u)}\right) . \end{aligned}$$

### Parametric approximation of hazards and switching between time scales

To investigate the impact of time scales, we will transform hazards from “time since hospital admission” to calendar time. For this, the hazards are being approximated parametrically in the time scale “time since hospital admission”. Dividing patients into two groups, we approximate the Nelson-Aalen estimator in each group by piecewise linear functions. The slopes of these approximations then provide piecewise constant approximations for the hazards in each group, based on the parametrization

4 $$\begin{aligned} \alpha _j(t) = \sum \limits _{k=1}^{K}\textbf{1}(t_{j,k-1} < t \le t_{j,k})\alpha _{j,k}, \ j \in \lbrace \textrm{1,2}\rbrace , \end{aligned}$$

with *t* the time since hospital admission, *K* the number of time intervals in which the hazards are constant, *j* the group indicator, $\alpha _{j,k}$ the constant value the hazard takes on in the time interval $(t_{j,k-1},t_k]$ and $0 = t_{j,0}< t_{j,1}< ... < t_{j,K}$ the cut points at which the hazard changes. The cut points and $\alpha _{j,k}$ can differ between groups.

The approximated hazards can be transformed from “time since hospital admission” to calendar time by calculating an individual hazard for each patient that depends on their hospital admission date and the number of days that have since passed, i.e.,

5 $$\begin{aligned} \bar{\alpha }_i(c) = \textbf{1}(c \ge c^*_i)\alpha _j(c - c^*_i), \ i = 1,...,n, \end{aligned}$$

where $\alpha _j(t)$ is the piecewise constant hazard in “time since hospital admission” of group *j* to which individual *i* belongs, *c* is a time point in calendar time and $c^*_i$ is the day of hospitalization of individual *i* in calendar time. Consequently, $c-c^*_i$ corresponds to the number of days that have passed since the hospital admission of patient *i*. Subsequently, an empirical parametric population hazard per group in calendar time can be calculated as the arithmetic mean of the hazards of all individuals in group *j* under observation at time point *c*, i.e.,

6 $$\begin{aligned} \tilde{\alpha }_j(c) = \frac{1}{\sum _{i=1}^n \tilde{Y}_{j,i}(c)}\sum \limits _{i=1}^n \tilde{Y}_{j,i}(c)\bar{\alpha }_i(c), \ c \ge c^*_{(1)}, \end{aligned}$$

with $\tilde{Y}_{j,i}(c) = \textbf{1}(\textrm{individual } i \in \textrm{group } j)\textbf{1}(c^*_i < c \le c^*_i + T^*_i)$ being the indicator of whether individual *i* belongs to group *j* and is at risk and under observation at time point *c*, $T^*_i$ the observed survival time of individual *i*, and $c^*_{(1)}$ the study entry of the first patient per group in calendar time.

From the two empirical parametric population hazards a time-dependent empirical parametric hazard ratio in calendar time can be calculated:

7 $$\begin{aligned} \tilde{\textrm{HR}}(c)=\frac{\tilde{\alpha }_{1}(c)}{\tilde{\alpha }_{2}(c)}. \end{aligned}$$

Note that the aim of approximations (4) - (7) is to investigate the empirical structural problem at hand.

### The Cox proportional hazards model

To investigate differences in mortality between groups, the Cox proportional hazards model was used. It models the hazard of an individual *i* as $\alpha (t|X_i) = \alpha _0(t) \exp (\beta X_i)$, where $\alpha _0(t)$ denotes the baseline hazard, which is the same for all individuals, and $X_i$ is the covariate. The aim is to estimate the unknown coefficient $\beta$. Under the proportional hazards model, it is assumed that the ratio of the hazards is constant over time. This assumption can, e.g., be checked with the test of Grambsch and Therneau [8].

Two Cox models were fitted, one defining time as “time since hospital admission” and one as “time since January 1^st^, 1970” (calendar time). Here, both regression models included only the certification group as a covariate, as this allows us to focus our analysis exclusively on the impact of the time scales on the significance of the estimated hazard ratio of the certification phases. To account for the potential correlation between patients in the same hospital, marginal Cox models were fitted [9].

#### Matching

Instead of using only the information of one time scale, one can use methods that account for both time scales. One option is a marginal Cox model using “time since hospital admission” as the main time scale while matching patients from different groups to have the same calendar date of admission. We used 1-to-1 matching, in which for each patient in the “Post” group, a matching partner was randomly selected from the patients in the “Pre” group who were admitted to a hospital on the same calendar date. The “Transitional” group was omitted. As a result, there is always one individual from the “Pre” group and one individual from the “Post” group with the same hospital admission date in the matched dataset. This assures comparability between the two groups “Pre” and “Post” with regard to the hospital admission dates, i.e., study entry times in calendar time, and resolves the structural bias caused by the use of calendar time. A matching partner was found for 3652 patients in the “Post” group. Two Cox regressions were fitted to the dataset containing only matched pairs, one for each time scale. As with the analysis on the full dataset, both regression models only included the certification phase as a covariate and were not adjusted for other variables to assess the direct impact of matching on the hazard ratios of the groups. The matching introduces a new correlation structure between subjects, which was again accounted for by fitting marginal Cox models [9].

#### Two time scales in the Cox proportional hazards model and the Poisson regression

Another option to account for two time scales is to include both in the Cox model by defining one time scale as the underlying time scale and including the other time scale as a covariate [3]. In the Cox regression with “time since hospital admission” as the underlying time scale, calendar time at the date of hospital admission was included as a baseline variable. For this, calendar time was transformed from days to years since January 1^st^, 1970, without loss of accuracy. In the Cox regression with calendar time as the underlying time scale, “time since hospital admission” in days was included as a time-varying covariate, which changed in weekly intervals. Weekly intervals were chosen because they are well interpretable and sufficiently compatible with the cut points used for the parametric approximation of hazards in the Impact of the time scale in the EvaCoM project section and the appendix. In addition, the computational costs are not too high. In both models, the time scale and the certification group were the only covariates.

However, in this approach, the time scales are treated differently. The underlying time scale is modelled non-parametrically, whereas the time scale that is included as a covariate is modelled parametrically [4, 10]. An alternative to the Cox model, which avoids this, is Poisson regression, as it allows both time scales to be incorporated simultaneously and models the impact of each time scale parametrically [4, 10, 11]. The Poisson regression has a close relation to the Cox model because the contribution of the follow-up of one person to the likelihood is equivalent to that of a Poisson-distributed observation, assuming a piecewise constant hazard [4, 10, 12, 13]. For the analysis of the EvaCoM data, we split “time since hospital admission” into four time intervals, as described in the Impact of the time scale in the EvaCoM project section and the appendix, but with the same interval limits for all groups. Calendar time was divided into annual intervals. In the Poisson regression, the only covariate was the certification group. Additionally, to account for the resulting time intervals having different durations, the logarithm of the length of the intervals was included in the model as an offset.

## Results

### Impact of the time scale in the EvaCoM project

The results from the Cox proportional hazard models in calendar time and “time since hospital admission” are shown in Table 1. The significance of the “Post” group versus the “Pre” group differs notably between the two Cox models. In both time scales, tests show no significant deviation from the proportional hazards assumption.

**Table 1:** The estimated hazard ratios (HR) with 95% confidence intervals (CI) and *p*-values for hazard of death of two marginal Cox models with different underlying time scales (“time since hospital admission” and “calendar time”). The group “Pre” served as the reference group

Underlying time scale	Certification phase	HR	CI	*P*-value
Time since hospital admission	Transitional	1.02	[0.95,1.09]	0.57
	Post	1.04	[0.99,1.10]	0.14
Calendar time	Transitional	1.05	[0.97,1.14]	0.20
	Post	1.13	[1.04,1.23]	0.01

**Fig. 1:**
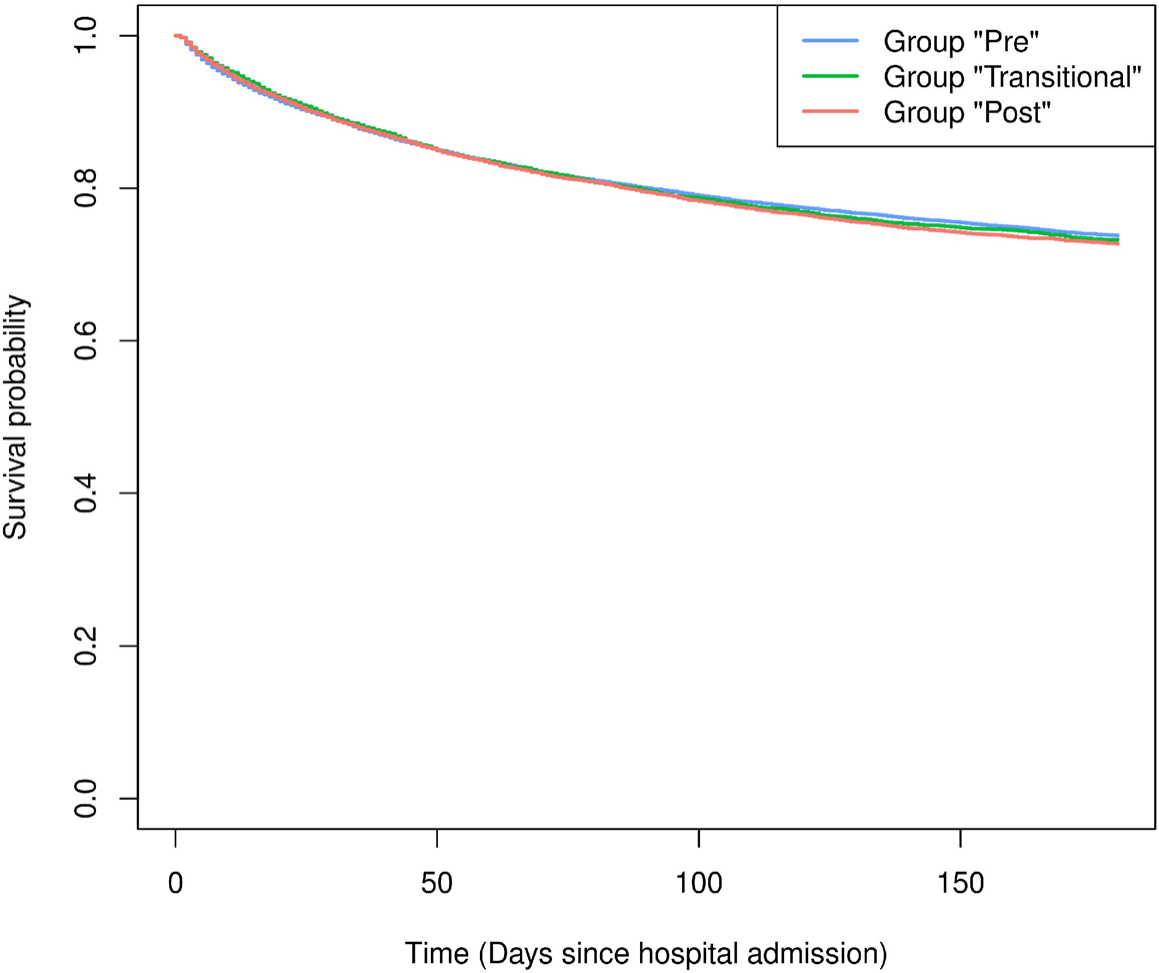
Kaplan-Meier estimators of the survival probabilities in the three groups “Pre”, “Transitional” and “Post” in the time scale “time since hospital admission”

The Kaplan-Meier estimators for the survival probabilities in “time since hospital admission” can be seen in Fig. 1. The estimated survival curves of the three groups are close together, which corresponds to the results of the Cox model (Table 1, top). Displaying the Kaplan-Meier estimators in calendar time on the other hand is not useful [3], because patients do not share one common time origin in calendar time.

**Fig. 2:**
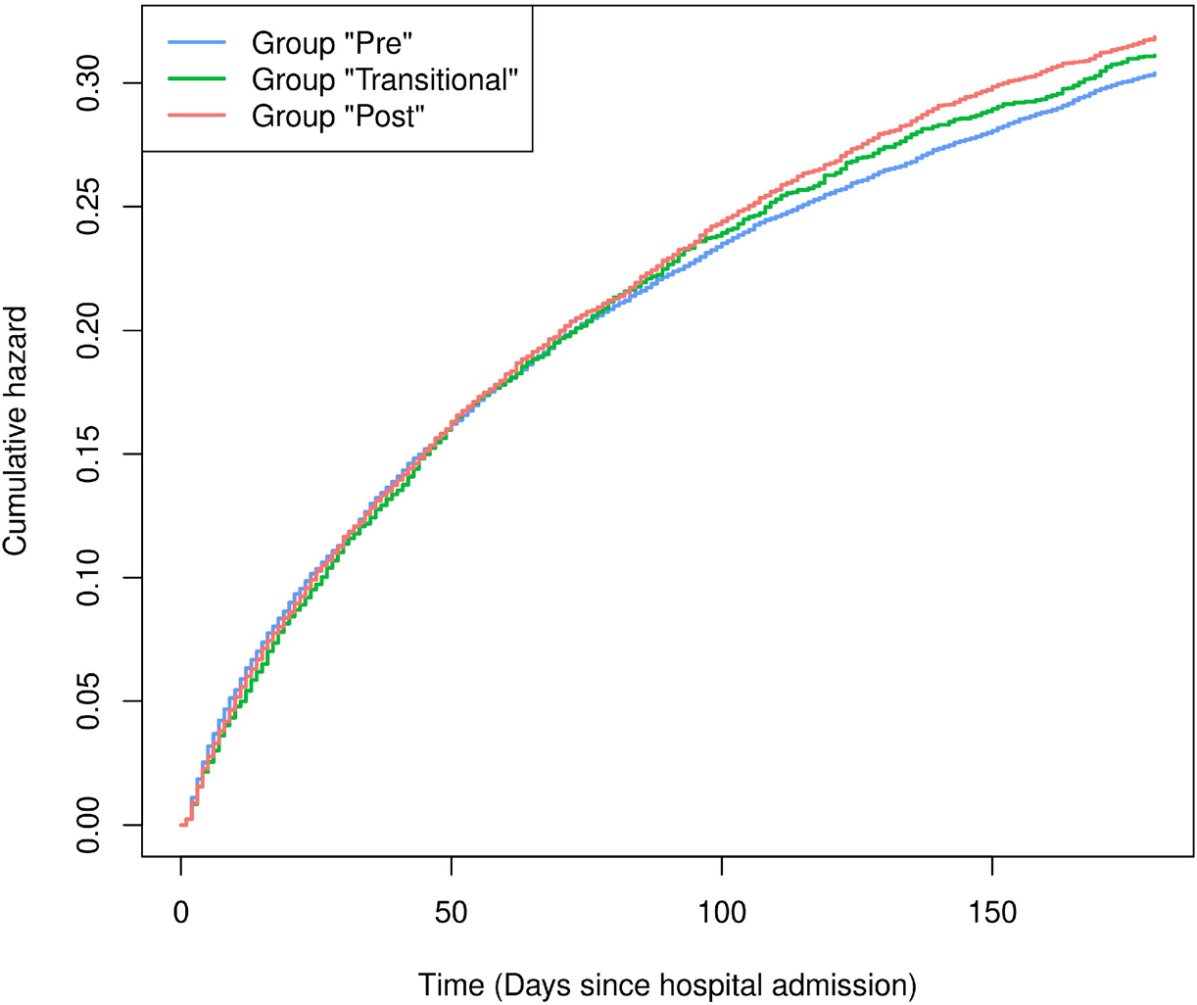
Nelson-Aalen estimators of the cumulative hazards for death in the three groups “Pre”, “Transitional” and “Post” in the time scale “time since hospital admission”

To investigate the contradictory results, we looked at the Nelson-Aalen estimators for the cumulative hazards in “time since hospital admission” for each group, which are shown in Fig. 2. The stratified Nelson-Aalen estimators in the certification groups are very similar, which also corresponds to the results of the Cox model (Table 1, top). Additionally, the slopes of the Nelson-Aalen estimators, i.e., the hazards, monotonically decrease. Thus, the hazard of dying is the highest in the first days after hospital admission and decreases thereafter. Considering the three groups “Pre”, “Transitional” and “Post” in calendar time, it is noticeable that due to their definitions, the groups are separated in time. Within a hospital, the study entries of patients in the “Pre” group always precede the entry times of patients in the “Transitional” group, which in turn precede the study entries of patients in the “Post” group. Therefore, at a time point in calendar time at which the groups can be compared, the hazards of the patients in the “Pre” group tend to be lower than those of patients from the other groups since they had more time to decrease.

The shift of the groups in calendar time and a flatter slope of the cumulative hazard in the “Pre” group than in the “Post” group at the first points in time at which both groups can be compared can also be seen in the Nelson-Aalen estimators in calendar time (Fig. B3 in the appendix). This supports our earlier impression that the contradictory results do not simply stem from a possible violation of the proportional hazard assumption, as the Nelson-Aalen estimator is a non-parametric estimator and does not rely on the proportional hazards assumption. Rather, the structural reason is illustrated as follows:

To visualize the impact of the shift of the groups in calendar time on the estimated hazard ratio, we approximated the Nelson-Aalen estimators in the groups “Pre” and “Post” in “time since hospital admission” with piecewise linear functions, which provided an estimator for piecewise constant hazards. The “Transitional” group was omitted for a more concise analysis without changing the definitions of the other two groups. Here, the assumption of piecewise constant hazards only serves the purpose of explaining the observed phenomenon, as it is a parametric model and therefore enables the transformations of hazards from one time scale to another [2]. For the parametric approximation, three cut points were chosen per group. These cut points and the piecewise constant hazards resulting from the parametric approximation are shown in the appendix.

**Fig. 3:**
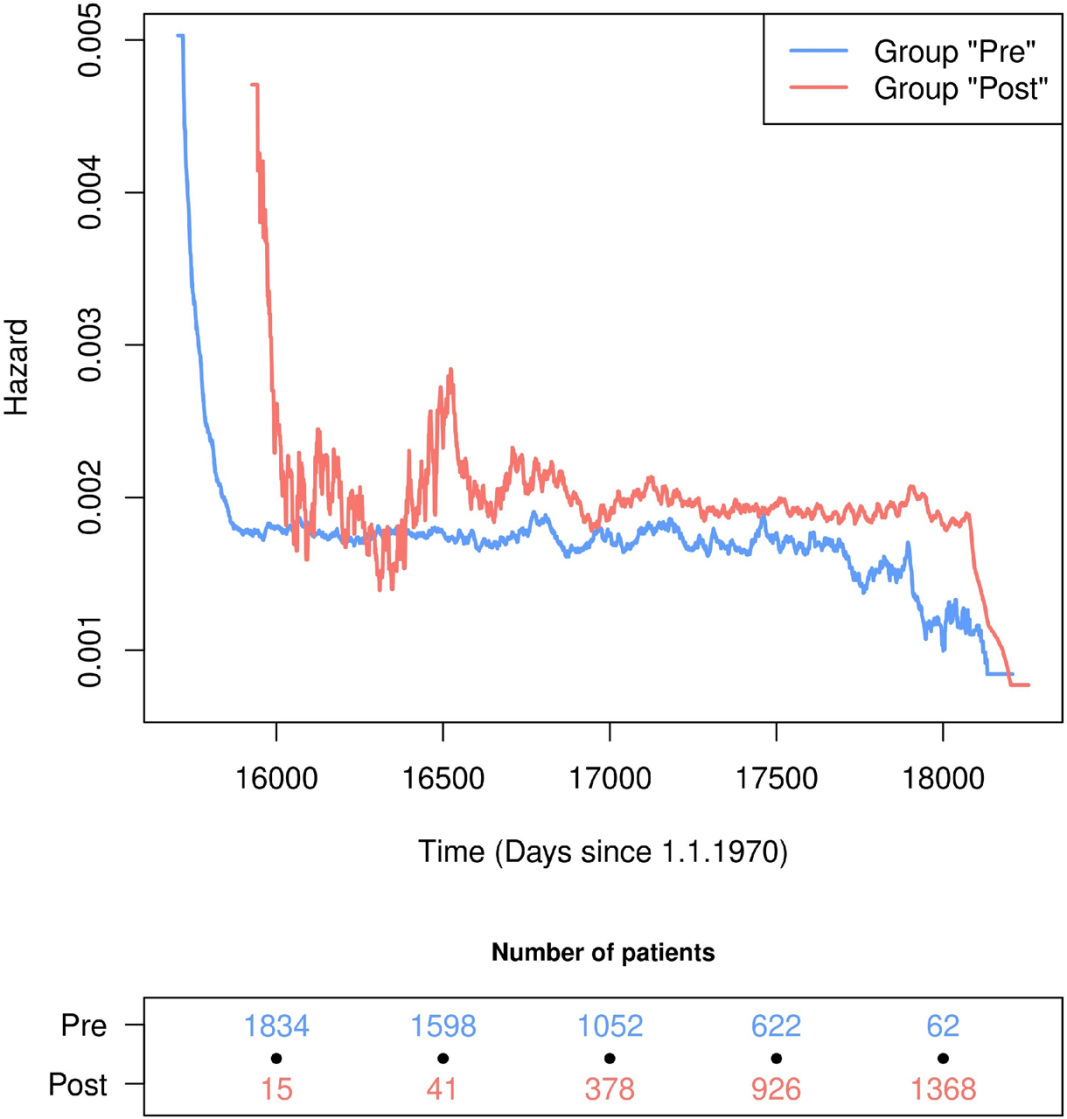
Parametric population hazards for death in calendar time for the groups “Pre” and “Post” obtained by transforming the piecewise constant approximated hazard from the time scale “time since hospital admission” to calendar time

The approximated hazards in “time since hospital admission” were then transformed into individual hazards in calendar time, and an empirical parametric population hazard in calendar time was calculated per group. These empirical parametric population hazards are shown in Fig. 3.

Our description of the hazards in calendar time finds that the two curves are not entirely different, but the population hazard of the “Pre” group decreases earlier than that of the “Post” group. Additionally, our description finds that the population hazard of the “Pre” group is lower at most time points. From time 16000 to time 16750, our empirical parametric approximation shows some spikes in the population hazard of the “Post” group. The reason for these spikes is the low patient numbers in this group due to left-truncation, which leads to uncertainties in the estimation [14, 15]. A check of whether the empirical parametric population hazards are good approximations of the hazards in calendar time can be found in the appendix.

**Fig. 4:**
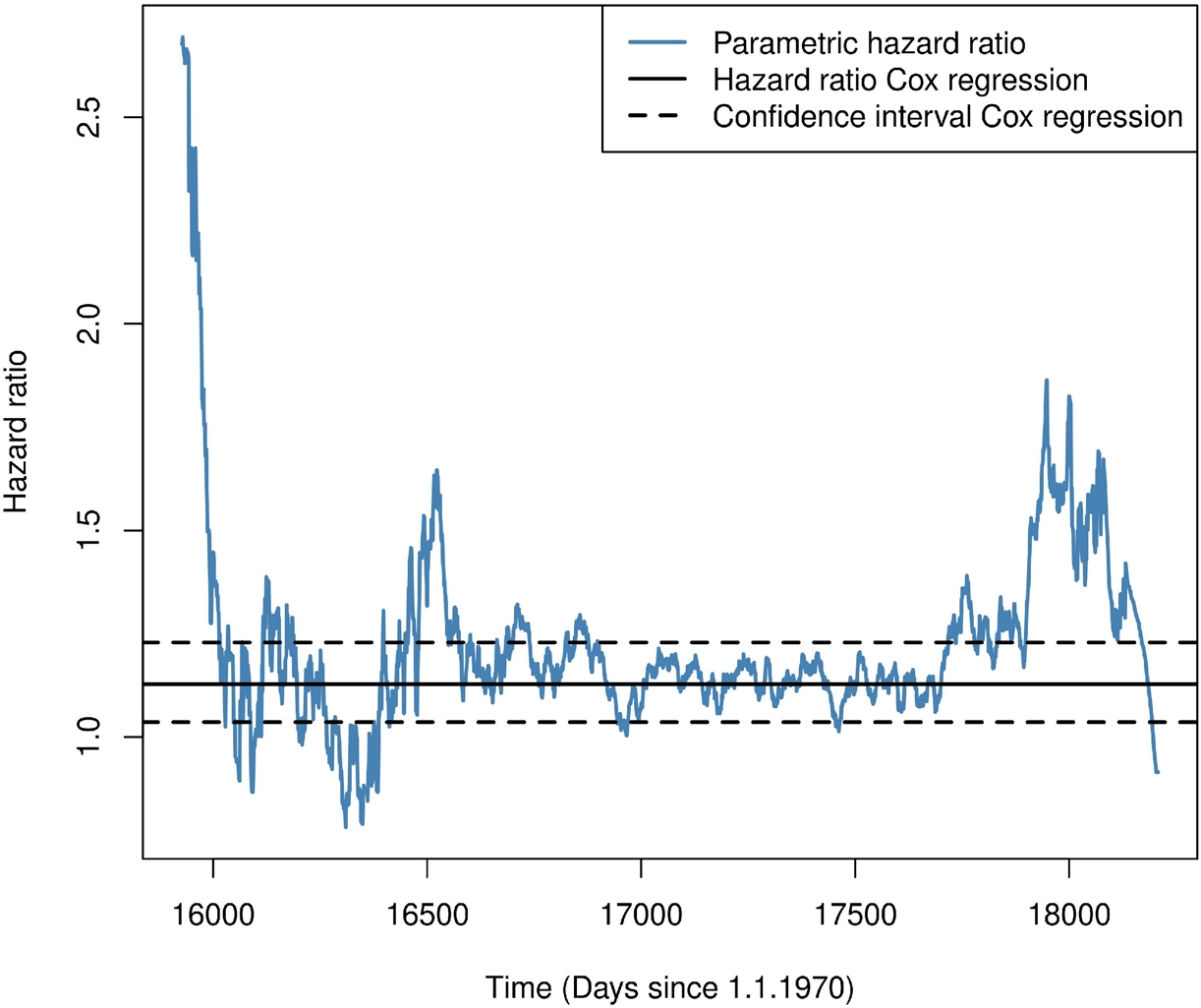
Comparison of the parametric hazard ratio for death with the hazard ratio and 95% confidence interval estimated by a marginal Cox model using “calendar time” as the underlying time scale

Since we are interested in investigating how the significant hazard ratio in calendar time arises, a time-dependent empirical parametric hazard ratio in calendar time was calculated using the two empirical parametric population hazards, which is shown in Fig. 4 together with the hazard ratio estimated by the Cox model in calendar time and its 95% confidence interval. Our empirical parametric approximation of the hazard ratio fluctuates around the hazard ratio estimated by the Cox model. Between 17000 and 17750 days since January 1^st^, 1970, the empirical parametric approximation lies within the 95% confidence interval estimated by the Cox model. The spikes in the empirical parametric approximation before and after this period stem from left-truncation and right-censoring, i.e., a low number of patients in one of the two groups. Overall, the parametric approximation is a useful descriptive tool that illustrates that the increase in the hazard for death in patients treated after certification to a trauma centre over the hazard in patients treated before the certification (Table 1 last row) is a consequence of the transformation to calendar time and the subsequent composition of the risk sets and not a true harmful effect.

We reiterate that we do not interpret Fig. 4 as illustrating a possible violation of the proportional hazards assumption as the reason for the seemingly contradictory results of Table 1. As noted above, the parametric curve is contained by the Cox confidence interval when there is a large number of patients in both groups. Our interpretation is that the parametric hazards model illustrates how to move from essentially no effect in patients’ time-to-event (Figs. 1 and 2) to a harmful effect in calendar time (Fig. 4).

### Resolving the estimation of an inflated hazard ratio in calendar time

The results from the matching approach are shown in Table 2. The matched Cox model in calendar time finds no significant difference between the groups and yields results similar to those of the Cox model in “time since hospital admission”.

**Table 2:** The estimated hazard ratios (HR) with 95% confidence intervals (CI) and *p*-values for hazard of death of two marginal Cox models on the dataset which included only matched pairs, as well as the estimates of the Poisson regression which incorporates both time scales simultaneously

Method	Underlying time scale	Certification phase	HR	CI	*P*-value
Matching	Time since hospital admission	Post	0.99	[0.91,1.09]	0.85
	Calendar time	Post	0.99	[0.90,1.09]	0.87
Cox with both time scales	Time since hospital admission	Transitional	0.99	[0.92,1.07]	0.81
		Post	1.00	[0.93,1.07]	0.99
	Calendar time	Transitional	0.99	[0.91,1.06]	0.70
		Post	1.00	[0.93,1.08]	0.90
Poisson		Transitional	0.99	[0.91,1.07]	0.77
		Post	1.00	[0.92,1.08]	0.94

The results from the regression models with two time scales can also be seen in Table 2. Both Cox models find no significant difference between the groups and estimate similar hazard ratios and confidence intervals. This is in line with the results of the Cox regressions on only matched pairs. The Poisson regression also does not find a significant difference between groups. A comparison of the results of all the approaches shows that even if the estimated hazard ratios and *p*-values are not exactly the same, they all have the same (non-)significance for the estimated parameters, and the estimates are very similar, too.

## Discussion

In this work, we analysed the association of certification to a specialised orthogeriatric centre with time-to-death on two time scales, either the patient’s time-to-event since hospital admission or calendar time, where the latter controls for time-varying factors affecting hospitals. In our example, the choice of time scale has a major impact on the significance of estimated hazard ratios, as the Cox model in calendar time estimated a harmful association of trauma centres with mortality, but the Cox model in “time since hospital admission” found no significant association. These seemingly contradicting results show how much of an impact the choice of time scale may have on subsequent analysis and why researchers should be careful when deciding which time scale to use. The choice of time scale is central in time-to-event analysis. While in randomized clinical trials, this choice is usually clear, it is often less obvious in cohort studies. Additionally, sometimes more than one time scale is assumed to influence probabilities. As a result, the choice of time scale is a topic that requires discussion [2, 4, 10]. The question of a meaningful “time 0” has additionally come into focus in the context of causal inference [16, 17]. Arjas [2] discusses the use of calendar time or “real time” as the underlying time scale and argues that the use of other time scales might change the natural sequencing of events in real time.

In the EvaCoM data, the contradictory results stem from the groups being shifted in calendar time and the fact that the hazards decrease after being admitted to a hospital. Therefore, it is important to check for potential pitfalls in the data with regard to time scales of interest. If, as in EvaCoM, the data structure in combination with a time scale leads to bias, one must find possible solutions. While time-on-study was a good choice here, this is not always the case, especially if the study entry starts only the follow-up and data collection but has no other contextual relevance [18].

One approach to resolve the estimation of significant hazard ratios due to the shift of groups in calendar time is matching. Here, 1-to-1 matching concerning the hospital admission date was used. This leads to a dataset in which the date of hospital admission is balanced between the groups. To account for the correlation between subjects due to matching, marginal Cox models were fitted [9]. A criticism of matching is that regression models can deal with confounding as effectively and that if the regression model is correctly specified, it has greater power than matching techniques do [19]. However, for the EvaCoM data, matching resolved the shift of groups in calendar time, as the two Cox models on the matched dataset estimated similar hazard ratios and *p*-values. Additionally, losing too many patients and subsequently power was not a concern here, as the dataset with only matched pairs still contained 7304 patients despite the strict 1-to-1 matching. A methodological restriction is that marginal matched Cox analysis has been developed for randomly right-censored data [9], but investigations on left-truncation as in calendar time are ongoing [20].

Additionally, we investigated the approach of accommodating more than one time scale in regression models. For the Cox model, one time scale is chosen as the underlying time scale, while the other is added as a covariate. This approach leads to the estimates of the Cox model in calendar time being similar to the estimates of the Cox model with “time since hospital admission” as the underlying time scale. However, the impact of the underlying time scale is modelled non-parametrically, whereas the impact of the time scale, which is incorporated as a covariate, is modelled parametrically [4, 10]. An alternative is the Poisson regression. It can accommodate multiple time scales simultaneously and therefore does not require to choose one underlying time scale [4, 10, 11]. Poisson regression has a close relation to the Cox model under the assumption of piecewise constant hazards [4, 10, 12, 13]. The Cox models using both time scales and the Poisson regression estimated similar hazard ratios and *p*-values. The fact that in our analysis treatment in trauma centres does not have a significant impact on the mortality of geriatric patients is in line with the findings of MacKenzie et al. [5], who found that patients treated in trauma centres in the United States have a lower overall risk of death than patients treated in other hospitals, but this effect to be not as pronounced in older patients, with the relative risks of death not differing significantly from 1.0 for older patients.

All regression models reported in this paper were not adjusted for potential confounders. Of course, adjusting the models is necessary in practice to meaningfully evaluate the association with the trauma centre. MacKenzie et al. [5] used propensity-score weighting to assure comparability of the patient populations of trauma centres and other hospitals. In our data, the phenomenon of contradictory results still occurred when the models were adjusted for patients’ age and sex, their care needs in the months before hospital admission, and their medication-based comorbidity score. So, the differences in significance do not stem from confounding issues, but from the shift of groups in calendar time. Therefore, in this paper only the unadjusted results were reported, as this allowed us to focus our analysis exclusively on the impact of the time scales on the significance of the estimated hazard ratio of the certification phases. Additionally, tests in both time scales show no significant deviation from the proportional hazards assumption, so the differences in significance were not attributed to obvious violations of this assumption.

In contrast to other EvaCoM analyses, the observation period from 2013 to 2021 was chosen here because this is a methodologically interesting period, in which, however, in part, only a few data are available for a few certified clinics and a few patients from certified clinics. If the calendar time is the only time scale used, a test shows a significant dependence of the event times on the left truncation. However, if the information of the time scale “time since hospital admission is taken into account in addition to calendar time, tests no longer show a significant dependence. The right-censoring, on the other hand, is solely administrative and therefore independent of the event times.

The results in the Resolving the estimation of inflated hazard ratio in calendar time section show that all three approaches seem to resolve the estimation of significant hazard ratios in calendar time. Here, the Cox regression in calendar time on the matched dataset works as well as the Cox regressions using both time scales and the Poisson regression. This makes matching a promising approach for resolving problems caused by a combination of data structure and a specific time scale, especially when only this time scale is of interest.

## Conclusions

The choice of time scale is often implicit in time-to-event analyses, but may severely impact results, as our data example from the EvaCoM project showed. In this work, we investigated the reason for the contradictory results of two Cox models in different time scales and presented methods to disentangle the effects of different time scales. The results indicate that the estimation of significant association in calendar time is not a true harmful effect but rather a consequence of the transformation to calendar time and the subsequent composition of risk sets. This shows that researchers must be careful when deciding which time scale to use and that it is important to check for potential pitfalls in the data with respect to time scales of interest.

## Supplementary Information

Additional file 1. The approximated piecewise constant hazards in the time scale “time since hospital admission” and a check of the transformation of the approximated hazards in “time since hospital admission” onto calendar time are provided in Additional file 1.pdf.

## Data Availability

The datasets supporting the conclusions of this article are owned by the German statutory health insurance AOK. Since public deposition of the data would breach ethical and legal compliance, data are only available upon formal request from the research institute of the AOK (WIdO). To request the data please contact the institutional body of the WIdO (wido@wido.bv.aok.de). To fulfill the legal requirements to obtain that kind of data, researchers must obtain permission for a specific research question from the German Federal (Social) Insurance Office. Additionally, researchers must conclude a contract with the statutory health insurance regarding data access which can be requested from the “AOK-Bundesverband GbR” (Federal Association of Local Health Insurance Funds) under http://aok-bv.De/kontakt/. The licensee is permitted to use the data for the purpose of the research proposal within their company, exclusively. Thereby, a company is defined as an economic unit. Licensees are not allowed to pass the data to a third party, or to create software or databases except for scientific publications. Moreover, the study has to be approved by the data protection officer both at the statutory health insurance and the research institute.

## Abbreviations

EvaCoM: Evaluation of orthogeriatric co-management for traumatological fractures in geriatric patients (Evaluation des unfallchirurgisch-geriatrischen Co-Managements bei alterstraumatologischen Frakturen älterer Patientinnen und Patienten)
DGU: German Society of Traumatology (Deutsche Gesellschaft für Unfallchirurgie)
DGG: German Society of Geriatrics (Deutsche Gesellschaft für Geriatrie)
WIdO: Research Institute of the AOK (Wissenschaftliches Institut der AOK)
HR: Hazard ratio
CI: Confidence interval
