# Supplementary material for: The choice of time scales in survival analysis has implications: calendar time versus patients’ time-to-event
Source: BMC Med Res Methodol. 2026 May 20;26:120. doi: 10.1186/s12874-026-02884-3 (PMC13192078; doi:10.1186/s12874-026-02884-3)
Supplement: Supplementary file 1 — Additional file 1. The approximated piecewise constant hazards in the time scale “time since hospital admission” and a check of the transformation of the approximated hazards in “time since hospital admission” onto calendar time are provided in Additional file 1.pdf. [file 12874_2026_2884_MOESM1_ESM.pdf]

The choice of time scales in survival analysis has implications: calendar time versus patients' time-to-event - Appendix

Appendix A Approximated piecewise constant hazards in the time scale "time since hospital admission"

Figure A1 shows the Nelson-Aalen estimators in the time scale "time since hospital admission" for the "Pre" and "Post" groups together with the curves resulting from their piecewise linear approximation. For the data of the EvaCoM project, three cut points were selected per group at which the piecewise constant hazard decreases, these cut points were $t_1 = 14.38$, $t_2 = 50.8$, and $t_3 = 102.61$ for the "Pre" group and $t_1 = 16.73$, $t_2 = 58.12$, and $t_3 = 125.08$ for the "Post" group. Figure A2 shows the piecewise constant hazards resulting from the piecewise linear approximation of the Nelson-Aalen estimators. One can see that the hazards are similar but, of course, not perfectly identical for the two groups.

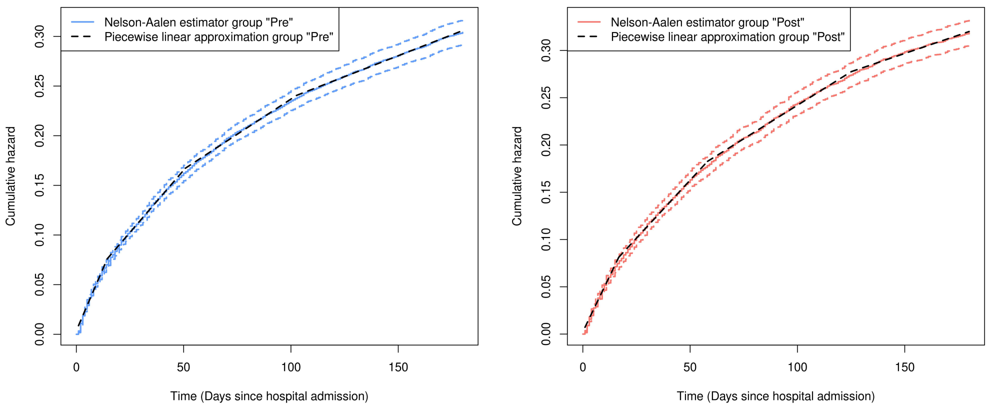

Fig. A1 Nelson-Aalen estimators of the cumulative hazards for death and their piecewise linear approximations for the groups "Pre" and "Post" in the time scale "time since hospital admission".

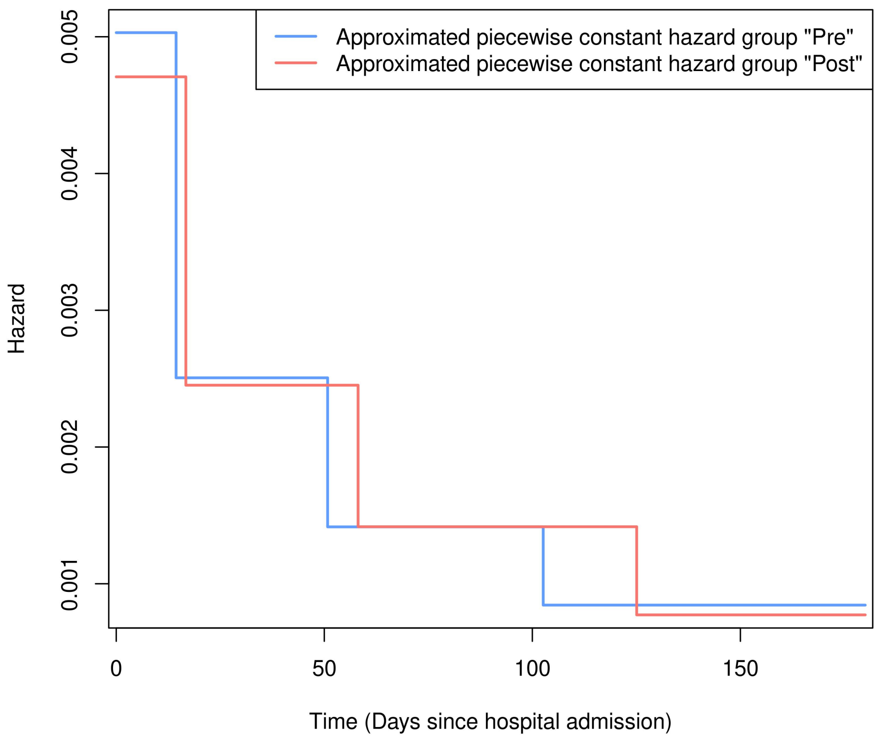

Fig. A2 Approximated piecewise constant hazards for death for the groups "Pre" and "Post" in the time scale "time since hospital admission".

Appendix B Check of the transformation of the approximated hazards in "time since hospital admission" onto calendar time

To check whether the transformation of the approximated hazards in "time since hospital admission" onto calendar time is a good approximation of the hazards in calendar time, we compared the Nelson-Aalen estimators in this time scale with the cumulative hazards calculated from the empirical parametric population hazards. The results are shown in Figure B3. Notably, if the left-truncation is dependent, the Nelson-Aalen estimator is not a consistent estimator for the cumulative hazard. However, since our parametric transformation replicates this possible dependency, a comparison is still meaningful.

For the "Pre" group the empirical parametric cumulative hazard is very close to the Nelson-Aalen estimator. For the "Post" group the empirical parametric cumulative hazard deviates from the Nelson-Aalen estimator but stays within its 95% confidence interval except for the first time points. The reason for this is the low patient numbers due to left-truncation. All in all, one can conclude, that the parametric approximation of the hazards in the time scale "time since hospital admission" and their transformation into calendar time works sufficiently well.

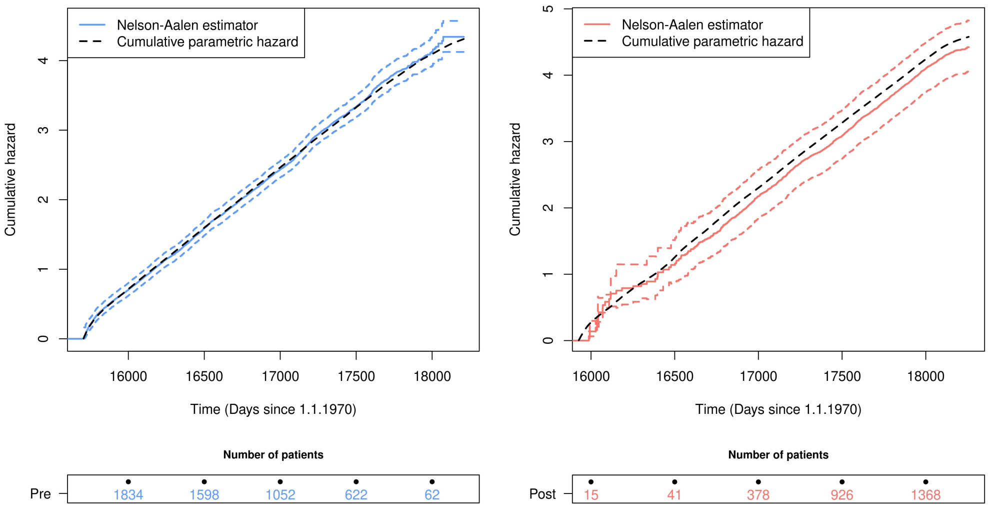

Fig. B3 Comparison of the cumulative empirical parametric population hazards for death with the Nelson-Aalen estimators of the cumulative hazards in the groups "Pre" (left-hand side) and "Post" (right-hand side) and their 95% confidence intervals in the time scale "calendar time".
